# Respiratory concerns of gabapentin and pregabalin: What does it mean to the pharmacovigilance systems in developing countries?

**DOI:** 10.12688/f1000research.21962.2

**Published:** 2021-02-25

**Authors:** Sunil Shrestha, Subish Palaian

**Affiliations:** 1 Department of Pharmacy, Nepal Cancer Hospital and Research Center, Lalitpur, Nepal; 2 Department of Pharmaceutical and Health Service Research, Nepal Health Research and Innovation Foundation, Lalitpur, Nepal; 3 Department of Clinical Sciences, College of Pharmacy and Health Sciences, Ajman University, Ajman, United Arab Emirates

**Keywords:** Gabapentin, Pregabalin, pharmacovigilance, Developing countries

## Abstract

Gabapentin and pregabalin, commonly known as gabapentinoids, have been widely used globally. This paper highlights the serious breathing problems due to using gabapentin and pregabalin which was warned by the United States Food and Drug Administration on December, 2019. In this article, we tried to recommend suggestions for controlling these adverse drug reactions (ADRs). Safety reports of gabapentin and pregabalin should be obtained from concerned manufacturers and reviewed for respiratory depression effects. There should be strict prescription monitoring and drug use evaluation studies. Concurrent use of gabapentin and pregabalin with other respiratory depressants such as opioids should be strictly monitored. Educating patients can help in the early detection of ADRs due to gabapentin and pregabalin. Anecdotal reports on these medications should be encouraged.

## Introduction

Gabapentin and pregabalin, commonly known as gabapentinoids, have been widely used globally. Gabapentin is an anticonvulsant agent used in treating various illnesses such as amyotrophic lateral sclerosis, analgesia, anxiety, neuralgia, restless legs syndrome and bipolar disorder ^1^. Pregabalin is commonly used to treat painful diabetic neuropathy, fibromyalgia, diabetic neuropathy, cancer chemotherapy-induced neuropathic pain, post-herpetic neuralgia, trigeminal neuralgia, and post-operative pain ^2, 3^. Pregabalin also acts to be an effective treatment therapy in refractory partial-onset seizures and the existing data recommends that pregabalin may be favorable as adjunctive therapy in adults with generalized or social anxiety disorder ^3^.

As per the available safety data, the use of gabapentin and pregabalin may cause neuropsychiatric related adverse drug reactions (ADRs) followed by hepatic, cutaneous and hematological reactions ^4^. Suicidal ideation, cognitive impairment, motor incoordination, dizziness are also severe forms of ADRs associated with gabapentinoids ^5^. The use of pregabalin is associated with hematological ADRs, and gabapentin is also associated with liver toxicity.

## Respiratory concerns with gabapentin and pregabalin

Respiratory depression, a highly mortal condition, due to gabapentin and pregabalin has been emerging for the past few years even in patients who were not on opioids, though post-marketing studies showed similar effects among patients taking these medications concurrently along with other respiratory suppressants ^6^. In December 2019, the United States Food and Drug Administration (US FDA) issued a drug safety alert on serious breathing problems with gabapentin and pregabalin noticed when used along with central nervous system (CNS) depressants or in patients with lung problems. US FDA reviewed data from the FDA Adverse Event Reporting System (FAERS) ^7^ database of almost five years, i.e. from January 1, 2012 – October 26, 2017, which revealed 49 cases gabapentinoid-induced respiratory depression. Out of 49, most cases (n=34; 69.3%) were reported with pregabalin and 30.6% (n=15) cases were reported with gabapentin. Of these cases, 92% reported either a respiratory risk factor, including age-related loss of lung function, or the use of a CNS depressant. This report also revealed that 24% percent (n=12) of the 49 patients with respiratory depression died due to respiratory depression and were taking gabapentinoids.

## What do the recent respiratory problems of gabapentin and pregabalin mean for pharmacovigilance in the developing world?

Since these ADR reports are from developed countries, it is difficult for regulators in developing countries to take decisions on these two medications that are also abundantly used in the developing world. For example, in India, there are escalating sales of gabapentin and pregabalin and while comparing the sales in 2017 to those in 2019, it was found that sales of gabapentin and pregabalin increased by 25% and 16%, respectively ^8^.

As is well documented in the literature ^9– 11^, pharmacovigilance programs in developing countries lack robust reporting of ADRs and underreporting is a common issue. To bring any regulatory changes such as labeling changes or even ultimately banning the medications, one needs evidence, which largely is lacking at this point among developing countries ^12^.

There have often been issues like this in the past where safety concerns emerge, mainly from the developed world, with drugs like selective cyclooxygenase-2 inhibitors (coxibs) ^13^, cerivastatin ^14^ and glitazones ^15^, and the developing world, due to lack of stringent pharmacovigilance mechanisms, is left with little or no choice but to follow the actions taken by the developed world. In addition, developing countries lack options for communicating pharmacovigilance information ^16^ among key stakeholders, including consumers, which is another concern. It is astonishing that many developing countries where large quantities of medicines are used still lack strong mechanisms to monitor the safety of their products.

## Recommendations

In the current scenario, safety reports of gabapentinoids should be obtained from concerned manufacturers and reviewed for respiratory depression effects. As understood, manufacturers are an important partner in the pharmacovigilance process with the unique advantage of formulation related information. The periodic safety update reviews (PSURs) submitted by the manufacturers of gabapentanoids can be an important source for new signal detection ^17^. In addition, the pharmaceutical manufacturer also has an obligation to report serious ADRs to the regulatory authorities ^18^.

Healthcare professionals should be watchful and report ADRs associated with gabapentinoids. Spontaneous reporting of suspected ADRs in the past have been crucial in detecting ADRs at an early stage ^19^.

Labeling changes should be made, and a drug can be banned if needed ^20^. New warnings are necessary to incorporate into prescribing information, including package inserts about possible respiratory depression ^8^. Concurrent use of these drugs with other respiratory depressants should be strictly monitored. Anecdotal reports on these medications should be encouraged as they can be crucial in detecting ADRs ^21^. If noticed, causality and severity assessments should be made for the suspected ADRs.

While these medications are used widely, it is also crucial to identify the vulnerable population at a high risk of developing respiratory side effects. It is evident that concurrent opioid use increases the risk for respiratory depression ^22^. Further, a retrospective study involving 175,787 patients who underwent colorectal surgery reported administration of gabapentinoids on the day of surgery was associated with an increased risk of pulmonary complications ^23^. In another retrospective analysis involving 858,306 patients who underwent total hip and knee arthroplasties, gabapentinoids showed respiratory suppression during the immediate post-operative period in a dose-dependent fashion ^24^. The patients on these two medications (and the ones closely chemically related to them) should be provided with proper counseling. Educating patients can help in the early detection of ADRs and active participation of patients can help identify adverse events and ADRs, describe ADRs, and ultimately prevent drug-related harm ^25^. Hospital drug and therapeutics committees play a crucial role in situations like this by disseminating information within the hospital ^26^. Social media may also play a crucial role in the signal generation of suspected ADRs in these situations ^27^. Hospitals using these medications should develop risk management plans associated with gabapentoinoids usage and must disseminate any safety issues to concerned authorities.

## Conclusions

Drug safety is a constantly evolving process and one must be vigilant on the use of these medications. It is suggested to exercise more caution while these drugs are used in patients and concurrent opioid administration. It is also essential to establish effective safety monitoring and reporting systems to identify signals and its proper dissemination. Healthcare professionals, especially prescribing physicians, nurses and pharmacists should be more cautious about using these medications in vulnerable people. Patient education and prescription restrictions of gabapentin and pregabalin may be needed until more data are available.

## Data availability

No data is associated with this article.
