# Supplementary material for: Respiratory concerns of gabapentin and pregabalin: What does it mean to the pharmacovigilance systems in developing countries?
Source: F1000Res. 2021 Feb 25;9:32. Originally published 2020 Jan 22. [Version 2] doi: 10.12688/f1000research.21962.2 (PMC7919607; doi:10.12688/f1000research.21962.2)
Supplement: Peer review history

# Peer review: Reviewer response for version 2

**DOI:** 10.5256/f1000research.54542.r80176

**Published:** 2021-02-26

**Authors:** Tetsu Ohnuma

**Affiliations:** 1 Department of Anesthesiology, Duke University Medical Center, Durham, North Carolina, USA

Thank you for addressing my points. I don't have further comments to make.

Is the topic of the opinion article discussed accurately in the context of the current literature?

Yes

Are arguments sufficiently supported by evidence from the published literature?

Partly

Are all factual statements correct and adequately supported by citations?

Partly

Are the conclusions drawn balanced and justified on the basis of the presented arguments?

Yes

Reviewer Expertise:

Perioperative multimodal analgesics

I confirm that I have read this submission and believe that I have an appropriate level of expertise to confirm that it is of an acceptable scientific standard.

# Peer review: Reviewer response for version 1

**DOI:** 10.5256/f1000research.24217.r68652

**Published:** 2020-08-17

**Authors:** Tetsu Ohnuma

**Affiliations:** 1 Department of Anesthesiology, Duke University Medical Center, Durham, North Carolina, USA

This is an interesting article on a relevant topic. It is well written and important points are clearly presented.

 Gabapentinoids, gabapentin and pregabalin, are being increasingly dispensed for indications outside US FDA labelling, particularly as part of multimodal analgesia in the perioperative setting. Emerging evidence from pre-clinical, human volunteer, inpatient, and outpatient studies suggests that their use in combination with opioids may increase the risk of respiratory depression.

 In our recent studies, use of gabapentinoids on the day of surgery was associated with increased risk of postoperative pulmonary complications in patients who underwent colorectal surgery ^1^. We also found that in-hospital use of both gabapentin and pregabalin had dose dependent associations with pulmonary complications in the immediate postoperative period after total hip and knee arthroplasties ^2^.

 The adverse effect of perioperative use of gabapentinoids can occur in all countries. While differences in risk across different populations are unclear (for example, the prevalence of pulmonary complications after concurrent use of opioids with gabapentinoids may differ among races), we suggest that a caution be warranted when these drugs are used in combination with opioids. Obviously, it is essential to establish effective safety reporting systems to detect signals of adverse events and disseminate the information to all relevant sections.

Is the topic of the opinion article discussed accurately in the context of the current literature?

Yes

Are arguments sufficiently supported by evidence from the published literature?

Partly

Are all factual statements correct and adequately supported by citations?

Partly

Are the conclusions drawn balanced and justified on the basis of the presented arguments?

Yes

Reviewer Expertise:

Perioperative multimodal analgesics

I confirm that I have read this submission and believe that I have an appropriate level of expertise to confirm that it is of an acceptable scientific standard, however I have significant reservations, as outlined above.

## References

[1] : Association 'Between Gabapentinoids on the Day of Colorectal Surgery and Adverse Postoperative Respiratory Outcomes. *Ann Surg*.270(6) : 10.1097/SLA.0000000000003317 e65-e67 10.1097/SLA.0000000000003317 30985370

[2] : Dose-Dependent Association of Gabapentinoids with Pulmonary Complications After Total Hip and Knee Arthroplasties. *J Bone Joint Surg Am*.2020;102(3) : 10.2106/JBJS.19.00889 221-229 10.2106/JBJS.19.00889 31804238

# Peer review: Reviewer response for version 1

**DOI:** 10.5256/f1000research.24217.r67201

**Published:** 2020-07-31

**Authors:** Sue E Jordan

**Affiliations:** 1 College of Human and Health Sciences, Swansea University, Swansea, UK

Thank you for the opportunity to review this interesting short opinion paper.

 The SmPCs for gabapentin and pregabalin include respiratory problems under ‘undesirable effects’; however, as the authors say, there are no recommendations to apply this to practice. This applies in all countries, particularly post-industrial societies. Our trial and observation study found too many unaddressed respiratory problems ^1^, ^2^.

 Whilst I concur with the sentiments expressed, I feel these should be placed in the context of full patient monitoring for the full range of possible adverse effects of prescribed medicines. Respiratory depression is extremely important, as indicated, but it is also important to check patients for other possible undesirable effects of medicines and unmedicated problems, including pain, sedation and falls. We have shown the effectiveness of this approach, and would like to see it more widely adopted ^1^, ^2^. Our website gives free access to our strategy. http://www.swansea.ac.uk/adre/.

Is the topic of the opinion article discussed accurately in the context of the current literature?

Yes

Are arguments sufficiently supported by evidence from the published literature?

Yes

Are all factual statements correct and adequately supported by citations?

Yes

Are the conclusions drawn balanced and justified on the basis of the presented arguments?

Yes

Reviewer Expertise:

NA

I confirm that I have read this submission and believe that I have an appropriate level of expertise to confirm that it is of an acceptable scientific standard.

## References

[1] : Nurse-Led Medicines' Monitoring for Patients with Dementia in Care Homes: A Pragmatic Cohort Stepped Wedge Cluster Randomised Trial. *PLoS One*.2015;10(10) : 10.1371/journal.pone.0140203 e0140203 10.1371/journal.pone.0140203 26461064PMC4603896

[2] : Nurse-led medicines' monitoring in care homes, implementing the Adverse Drug Reaction (ADRe) Profile improvement initiative for mental health medicines: An observational and interview study. *PLoS One*.2019;14(9) : 10.1371/journal.pone.0220885 e0220885 10.1371/journal.pone.0220885 31509537PMC6738583
